# Imaging in Large Vessel Vasculitis—A Narrative Review

**DOI:** 10.3390/jcm13216364

**Published:** 2024-10-24

**Authors:** Ioana Popescu, Roxana Pintican, Luminita Cocarla, Benjamin Burger, Irina Sandu, George Popa, Alexandra Dadarlat, Raluca Rancea, Alexandru Oprea, Alexandru Goicea, Laura Damian, Alexandru Manea, Ruben Mateas, Simona Manole

**Affiliations:** 1 Department of Radiology, County Emergency Hospital, 400006 Cluj-Napoca, Romania; ioanapopescu1014@gmail.com; 2 Radiology Department, “Iuliu Hatieganu” University of Medicine and Pharmacy, 400337 Cluj-Napoca, Romania; 3 Department of Radiology, “Prof. Dr. Ion Chiricuta” Oncology Institute, 400015 Cluj-Napoca, Romania; 4 Department of Radiology, “Niculae Stancioiu” Heart Institute, 400001 Cluj-Napoca, Romania; luminita.cocarla@institutulinimii.ro (L.C.); benjamin.burger@institutulinimii.ro (B.B.); irina.sandu@institutulinimii.ro (I.S.); george.popa@institutulinimii.ro (G.P.); 5 Department of Cardiology, “Niculae Stancioiu” Heart Institute, 400001 Cluj-Napoca, Romania; dadarlat.alexandra@umfcluj.ro (A.D.); rancea.raluca@elearn.umfcluj.ro (R.R.); 6 Cardiology Department, “Iuliu Hatieganu” University of Medicine and Pharmacy, 400337 Cluj-Napoca, Romania; 7 Cardiothoracic Surgery Department, “Iuliu Hatieganu” University of Medicine and Pharmacy, 400337 Cluj-Napoca, Romania; alexandru.oprea@elearn.umfcluj.ro (A.O.); goicea.alexandru@elearn.umfcluj.ro (A.G.); manea.alexandru@elearn.umfcluj.ro (A.M.); mateas.ruben@elearn.umfcluj.ro (R.M.); 8 Department of Cardiovascular Surgery, “Niculae Stancioiu” Heart Institute, 400001 Cluj-Napoca, Romania; 9 Department of Rheumatology, County Emergency Hospital, 400006 Cluj-Napoca, Romania; ldamian.reumatologie@gmail.com

**Keywords:** large vessel vasculitis, giant cell arteritis, Takayasu arteritis, CTA

## Abstract

Vasculitis refers to a group of rare conditions characterized by the inflammation of blood vessels, affecting multiple systems. It presents a diagnostic and therapeutic challenge due to its broad clinical manifestations. Vasculitis is classified based on the size of the affected vessels: small, medium, large, or variable-sized. Large vessel vasculitis (LVV), particularly giant cell arteritis (GCA) and Takayasu arteritis (TAK), has garnered attention due to its significant morbidity and mortality. Both conditions involve immune-mediated inflammation of the vascular wall, despite differing in epidemiology and presentation. Early identification is crucial to prevent complications like organ ischemia and hemorrhage. Diagnostic accuracy can be hampered by false negative results, making comprehensive investigation essential. Vascular imaging, including computed tomography angiography (CTA), ultrasound (US), magnetic resonance imaging (MRI), and positron emission tomography-computed tomography (PET-CT), is key in diagnosing vasculitis, revealing vessel wall thickening and other suggestive features. This article reviews typical and atypical CT and CTA findings in LVV, discusses imaging modalities, and highlights their role in therapeutic management and prognosis. It emphasizes the importance of a multidisciplinary approach and the critical role of radiologists in improving patient outcomes in LVV.

## 1. Introduction

Vasculitis describes a heterogenous group of rare conditions that are characterized by histologic evidence of multisystemic blood vessel inflammation [1]. Large vessel vasculitis (LVV) has been the main topic of many recent studies, given its potential for significant morbidity and mortality. LVV comprises two main entities, giant cell arteritis (GCA) and Takayasu arteritis (TAK). GCA is the most common primary vasculitis, with high global variation in incidence and estimates of 44 cases in 100,000 persons above 50 years of age in Northern Europe [2]. TAK has an annual incidence of 0.4–3.4 cases in 1,000,000 persons [2]. Similarly, other conditions can potentially involve large and pulmonary vessels, such as Hughes–Stovin syndrome (HSS) and Behcet’s disease (BD).

Blood vessel stenoses and occlusions with subsequent organ ischemia, as well as aneurysm formation or hemorrhage, are serious complications of vasculitis [1]. Thus, prompt identification is of great importance given the increased risk of mortality. With the advances made in the field of radiology, vascular imaging has become a great tool in the diagnosis of vasculitis, alongside histopathological and immunofluorescence studies [3]. Computed tomography angiography (CTA) along with ultrasound (US), magnetic resonance imaging (MRI), and positron emission tomography-computed tomography (PET-CT) play important roles in the diagnosis of LVV, as well as in the assessment of the extent and severity [2].

The broad spectrum of clinical manifestations, frequently non-specific, as well as the high rates of complications have made vasculitis a diagnostic and therapeutic challenge to clinicians throughout the world. This article aims to provide an overview of large vessel vasculitis from a radiological perspective, encompassing the diagnostic radiological modalities and findings, with CT/CTA imaging as the main focus, as well as underline the importance of a multidisciplinary approach in the management of these conditions. Consequently, we seek to facilitate the understanding of LVV and improve diagnostic accuracy among radiologists by sensitizing them to both the typical radiological findings as well as the differential diagnoses and mimickers of these pathologies.

## 2. Classification and Nomenclature

Considering the heterogenous and large spectrum of vasculitis, the need to reach a consensus on names for the most common forms of vasculitis and to construct a specific definition for each was needed. Hence, the Chapel Hill International Consensus Conferences (CHCC) held in 1994 and 2012 defined and standardized the nomenclature of systemic vasculitis, which is summarized in Table 1. The CHCC is a nomenclature system, not a classification or diagnostic system, that directs the management of vasculitis [4].

The complex pathophysiology involved and the diverse manifestations of vasculitides led to the proposal of many classification criteria.

Vasculitides are universally divided according to the diameter of the main vessels that are involved: “small vessel” (including arterioles, capillaries, and postcapillary venules); “medium vessel” (referring to the main visceral vessels and their initial branches); “large vessel” (including the aorta and its major branches). The term “variable vessel disease” (VVV) has been used in reference to a group of conditions that can affect multiple vessels of different calibers, including large vessels, the most common conditions being Behçet’s disease and Cogan syndrome [3]. Hughes–Stovin syndrome (HSS) is a very rare disorder, an LVV with many similarities to Behçet’s disease, also referred to as “incomplete Behçet’s disease” [5].

Overlapping of these categories is not uncommon, and the combined involvement of different caliber vessels can be seen. In this case, the dominant vessel involvement leads the diagnosis. On the other hand, single-organ vasculitis has been described, with isolated aortitis being the main representative of this group [6].

From a clinical and therapeutical standpoint, vasculitides can be broadly divided into two categories: primary and secondary. Primary (idiopathic) vasculitides include a spectrum of non-infectious disease entities that have unique clinical, histopathologic, and therapeutic characteristics [1]. On the other hand, secondary vasculitides can be associated with systemic disease (systemic lupus erythematosus, rheumatoid arthritis, sarcoidosis, and IgG4-RD) or other probable specific causes (hepatitis B and C, syphilis, drugs, and malignancies). Usually incidentally detected during clinical workup, secondary vasculitides may be accompanied by symptoms and complications of the primary disease and require specific treatment of the underlying cause [1,7].

## 3. Epidemiology

Vasculitis is more prevalent in adulthood; the mean age of diagnosis in adults is 47 years old. However, several vasculitides can be diagnosed at any age, with a mean age of diagnosis in children 7 years old [3].

Amongst systemic large vessel vasculitis (LVV), giant cell arteritis (GCA) is the most common form, with diagnosis being made almost exclusively in patients older than 50 years and twice as often in females as in males [1,6]. GCA shows a predominance in people of European ancestry, with the highest rates in northern countries such as Sweden and Norway (over 20 new cases of per year per 100,000 individuals over 50 years of age) [6].

Takayasu arteritis (TAK) has been mostly observed in Asia (with 40 cases per 1,000,000 people), although it can be seen throughout the world. Higher rates have been described in women; however, a higher incidence of hypertension and abdominal aorta involvement is seen in male patients [1,6].

Amongst the variable vessel vasculitides that can affect the large-caliber vessels, Behçet’s disease (BD) occurs in younger people of Asian and Eastern Mediterranean descent, mostly in males between the ages of 20 and 35 years [1]. Prevalence rates vary, ranging from 1.5–16 per 100,000 individuals in southern Europe to 0.3–5 in northern Europe [6].

## 4. Pathophysiology and Genetics

GCA, also known in the past as temporal or Horton arteritis, is a granulomatous vasculitis that predominantly involves the extracranial carotid branches [1]. Classically, the main pathological changes in GCA are inflammation of the arterial wall, fragmentation of the internal elastic lamina, and thickening of the intima. Arterial involvement may be focal or segmental, creating the appearance of “skip lesions” [8]. In GCA, four phenotypes have been described, which can occur individually, concomitantly, or sequentially: LVV, cranial disease, polymyalgia rheumatica, and systemic inflammatory disease [1]. Studies have shown powerful associations between GCA and human leukocyte antigen HLA-DRB*04 alleles and the involvement of gene polymorphisms such as PTPN22, NOS2, ERAP1, REL, and PRKQC [2,6,9].

TAK is characterized by variable degrees of inflammation (acute exudative, chronic, and granulomatous) of all arterial layers (panarteritis), mainly the media and adventitia, associated with hyperplasia and neovascularization of the intimal layer. In longstanding disease, these changes eventually lead to fibrosis of the arterial wall [6]. In the context of TAK, a persistent genetic risk factor associated in multiple cohorts of different ethnicities is HLA-B5201, confirmed in genome scanning studies [2,9]. Two independent susceptibility loci in the HLA region were identified and confirmed: HLA-B/MICA and HLA-DQB1/HLA-DRB1 [6].

In BD, no clear etiology has been confirmed and it is mainly considered an auto-inflammatory systemic vasculitis [10]. The major associated signals are located within the MHC class I region, with HLA-B51 alleles being the primary genetic susceptibility factor, consistently linked with BD in different ethnicities [9]. The possibility of microbial infection involved in the pathogenesis was raised, even though BD is not a classical infectious disease [10].

## 5. Clinical Presentation

Giant cell arteritis (GCA) typically develops gradually over weeks to months, though in about 20% of cases, the onset can be sudden [8]. GCA often presents with cranial artery involvement and constitutional symptoms such as headache, jaw or tongue pain during chewing, scalp tenderness, unexplained weight loss, and fever [1,6,8]. Polymyalgia rheumatica, which manifests as aching and stiffness in the shoulders and hips, is present in 40–60% of individuals with GCA [1,6]. Involvement of the aorta or its main branches occurs in 27% of cases and can cause limb claudication [1].

Vascular occlusion is the main cause of cranial ischemic events in GCA patients, with ischemic optic neuropathy being the most dreaded complication, which can occur in 14% of patients [1,6,8]. Vision loss, which can be partial or complete and may affect one or both eyes, is typically painless and irreversible once it occurs [6,8]. Other ischemic complications of GCA caused by hypercoagulability, accelerated atherosclerosis, and direct endothelial damage, include transient ischemic attacks and strokes, which occur in 2% to 7% of cases and represent the leading cause of mortality in these patients [6].

Takayasu arteritis (TAK) may present with a range of clinical manifestations from more general constitutional or inflammatory symptoms without evident signs of arterial occlusion (pre-pulseless stage) to vascular symptoms caused by arteritis (pulseless stage) [6]. Systemic symptoms include fatigue, general discomfort, weight loss, night sweats, fever, joint pain, and muscle aches [1].

Vascular symptoms depend on the location, type of lesion, and the adequacy of collateral blood flow [1]. The disease can affect the entire aorta, with the subclavian and common carotid arteries being the most involved branches. Stenotic lesions are most prevalent, occurring in over 90% of cases, while aneurysms are observed in approximately 25% of patients. [6]. TAK is the primary cause of aortitis in younger individuals [6]. Hypertension affects 30–90% of patients and often leads to renal, cardiac, and cerebral complications [1]. Strokes and transient ischemic attacks occur in an estimated 10% to 20% of patients [6]. Stenosis of the renal artery is seen in 20–30% of patients with TAK and hypertension. Ischemic retinopathy affects approximately 14% of TAK patients. Cardiovascular events such as heart failure, myocardial infarction, pulmonary hypertension, and even ruptured aortic aneurysms are concerning sources of mortality [6].

TAK can also be linked with other inflammatory conditions, including sarcoidosis, spondylarthritis, and Crohn’s disease [6].

Behcet’s disease is usually associated with recurrent aphthous oral ulcers and at least two of the following: genital ulcerations, eye lesions (uveitis), skin lesions (pseudofolliculitis and erythema nodosum), or a positive pathergy test [4,6]. More severe manifestations include gastrointestinal inflammation and ulcerations, blindness, meningoencephalitis, and vascular complications [4].

Up to 40% of patients may experience large vascular issues [4], including thrombosis, vessel occlusion, or aneurysms in the pulmonary or peripheral arteries [4,6].

The disease can be fatal in about 4% of cases, typically due to gastrointestinal perforation, vascular rupture, or central nervous system complications [4]. Remissions and relapses are frequent [4].

## 6. Imaging Findings

### 6.1. Imaging Modalities

Imaging is essential for both the diagnosis and management of LVV, especially considering the difficulties of performing tissue biopsies on large vessels. While traditional methods such as US, Doppler-US, CTA, or MRA (magnetic resonance angiography) mainly focus on assessing vessel structure, there is increasing interest in using functional and molecular imaging techniques like F-fluorodeoxyglucose (FDG) PET/CT. Invasive imaging methods, previously used for LVV diagnosis, are now mainly reserved for treatment interventions, such as angioplasty or stenting, because they are not as effective in evaluating vessel wall integrity [7].

All of the different imaging modalities used in the diagnosis of LVV have both advantages and disadvantages. The right examination should be chosen based on the particularities of the cases and the limitations they impose [11]. Table 2 exemplifies the main advantages and disadvantages of US, CTA, MRA, and PT/CT in the assessment of LVV.

CTA facilitates vessel evaluation with excellent spatial resolution and widespread availability. It allows for extensive assessment of the entire aorta and its major branches. The preferred approach for arterial imaging involves a triphasic image acquisition, comprising unenhanced images, ECG-gated arterial phase, and an additional delayed phase to assess arterial parietal enhancement [7,13].

CTA examinations of vasculitis should follow the recommended protocol:

- Multislice CT scanner is preferred;
- Collimation should be 0.6 mm, tube should be 120 kV;
- Tube current time product (mAs) should be determined by automatic dose modulation;
- Reconstruction slice thickness should be 0.5–1.0 mm;
- Injection should be adapted to the bodyweight: 60–120 mL of non-ionic iodinated contrast agent (>/=350 mg/mL) using a power injector (>/=4 mL/s);
- Arterial phase: bolus tracking method (threshold of 100 HU), ECG triggering;
- Venous phase: acquisition 50 s after finishing the arterial phase;
- Additional delayed phase—optional;
- Angulated reconstructions for assessment of individual vessel segments [12,14].

One prospective study revealed that the sensitivity and specificity of CTA in detecting mural edema seen early in the disease process were 73% and 78%, respectively [15].

### 6.2. Imaging Findings in Primary LVV

Radiological assessment of LVV can be performed using different imaging techniques depending on their accessibility, contraindications associated with every patient, the presumed findings, and the location of the vascular changes. Table 3 lists the main imaging features detected using US, CTA, MR, and PET/CT [7].

Due to CTA’s high spatial resolution and capability of assessing large vessel wall abnormalities with a single acquisition, as well as its wide availability [7], our work will focus mainly on the radiological examination of LVV using computed tomography.

Classically, the radiological appearance of LVV on CTA consists of circumferential vessel wall thickening of over 2–3 mm and parietal enhancement. Arterial wall enhancement, often difficult to assess, is defined as an attenuation increase of more than 20 Hounsfield units compared to the wall attenuation on unenhanced images and can be emphasized by delayed scanning. Perivascular fat stranding can sometimes be present [7,13].

#### 6.2.1. Giant Cell Arteritis

In GCA, large vessel involvement occurs in less than half of the cases, in about 27% of diagnosed patients [1]. The aorta is affected in 45–65% of patients with large vessel GCA, the thoracic aorta being involved in the majority of those cases, while distal lesions of the subclavian, axillary, and brachial arteries have variable occurrence [8,13]. Rarely, lower extremity arteries are affected [8]. Involvement of the temporal artery is generally assessed using US or contrast-enhanced high-resolution MRI [8].

The radiological report is important in defining the stage of the disease and ultimately in establishing the correct treatment. The key feature of GCA is the transmural inflammation of large elastic arteries. Thickening of the arterial wall assessed as over 2 mm for the aorta and over 1 mm for branch vessels [13] on CTA or MRI suggests vessel wall inflammation and is directly indicative of active disease [5,16,17]. In longstanding disease, this leads to mural hyperplasia and subsequent long stenotic segments with luminal occlusion [17].

**Figure 1:**
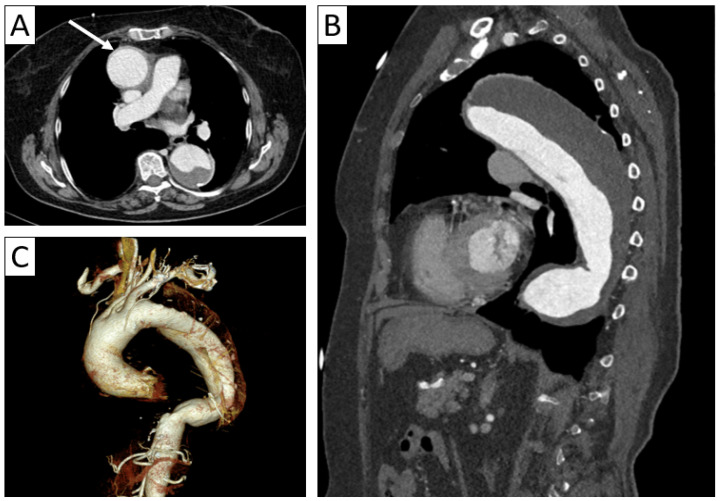
An 80-year-old female patient with giant cell arteritis and rheumatoid arthritis. Axial thoracic CTA (**A**) with sagittal reformatting (**B**) and 3D volume rendering (**C**) demonstrated minimal thickening of the aortic wall (arrow in (**A**)) and a fusiform aneurysm of the ascending and descending thoracic aorta with parietal thrombosis.

The main complications of GCA are narrowing and obstruction of the arteries leading to ischemic events. Additionally, the loss of structural integrity of the medial layer of the vessel wall leads to ectasias and aneurysm formation, less frequent in GCA compared to TAK [18]. The “string of pearls” sign can be identified in vessels that present with both ectasias and stenotic segments [17]. A cross-sectional study using CT revealed that over 20% of the patients with GCA (12 out of 54 patients included in the study) developed aortic aneurysms after 4 to 10 years, with an associated relative risk of 3–17 [19] (Figure 1). Patients with GCA have a higher aneurysmal growth rate in comparison to patients with degenerative disease and a higher risk of aortic dissection at a smaller aortic caliber of under 50 mm [13].

Other potentially life-threatening complications of GCA include aortic insufficiency, acute arterial dissection, rupture, and hemorrhage [5,17,20].

#### 6.2.2. Takayasu Arteritis

TAK primarily affects the great vessels (aorta and its major branches with a prevalence of 65%), as well as the coronary (44%) and pulmonary arteries (63%) [21].

Angiographically, based on the aortic involvement sites, 6 types of TAK have been distinguished: type I—the aortic arch; type IIa—the ascending aorta, aortic arch, and its branches; type IIb—the ascending aorta, aortic arch and its branches, and the descending thoracic aorta; type III—the descending thoracic aorta, abdominal aorta, or the renal arteries; type IV—the abdominal aorta or renal arteries; and type V—all aortic segments [6].

Based on the duration of ongoing disease, differentiation of acute and chronic stages is important for future management.

**Figure 2:**
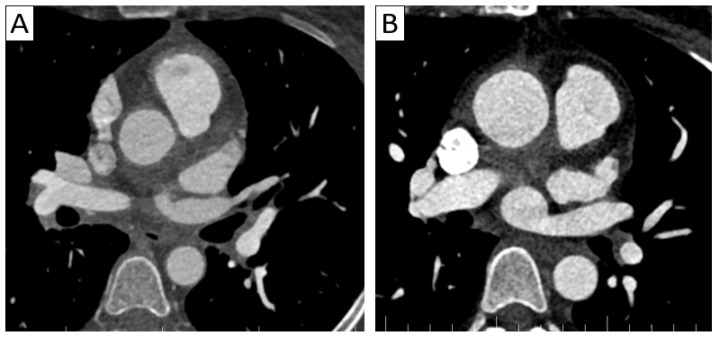
(**A**) A 37-year-old patient with Takayasu arteritis presenting with unstable angina. Axial thoracic CTA demonstrated aortic wall thickening and periaortic infiltration, as well as parietal inflammation of the supra-aortic trunks and coronary arteries, with myocardial infarction in the left anterior descending coronary artery territory. (**B**) A 35-year-old patient with Takayasu arteritis presenting with severe aortic insufficiency—aortitis was suspicioned intraoperatively during valvular replacement. Axial thoracic CTA demonstrated parietal thickening of the ascending aorta and periaortic infiltration.

**Figure 3:**
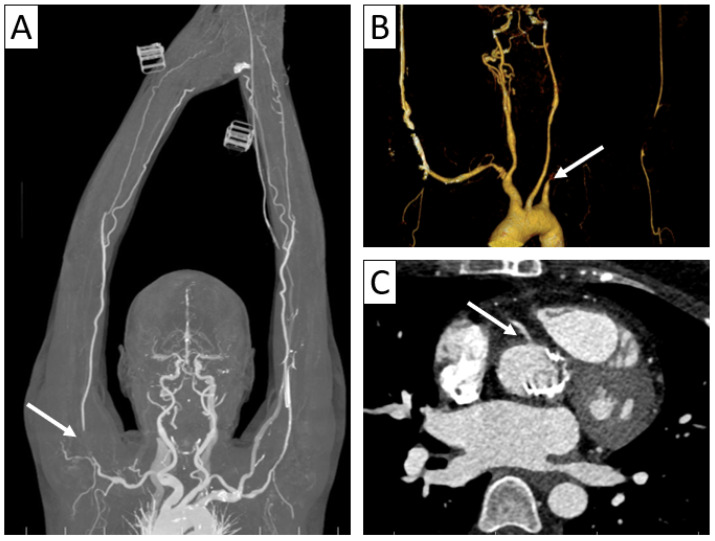
Complications of vasculitis—stenoses and occlusions. (**A**) Maximum intensity projection (MIP) of 3D-CTA showed brachial sub-occlusion (arrow) in a patient with parietal thickening of up to 4 mm in the supra-aortic trunks with extension to the axillary and brachial arteries. (**B**) Supra-aortic trunk CTA 3D volume rendering in a 38-year-old patient with TAK and Behcet’s disease overlap showed occlusion of the left subclavian and axillary arteries (arrow) with weak brachial artery distal reperfusion. (**C**) Axial cardiac CT in a 35-year-old patient with TAK revealing aortic wall thickening extending to the right coronary artery ostium with minor stenosis (arrow).

**Figure 4:**
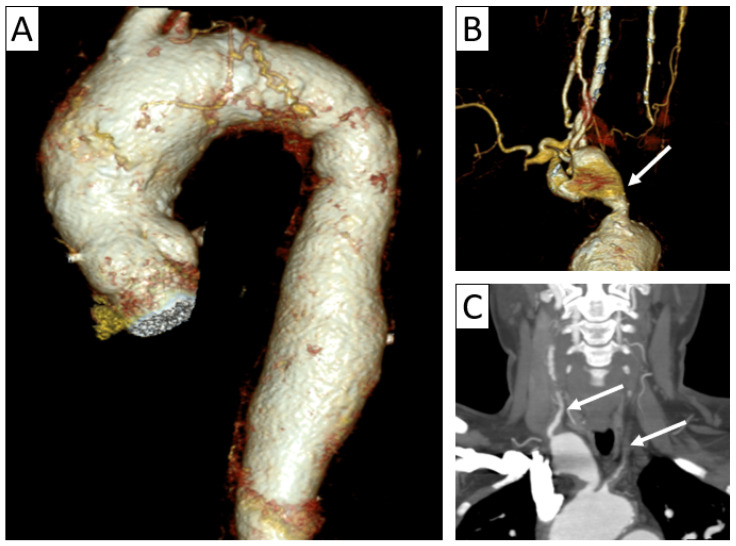
Complications of vasculitis—stenoses and aneurysms. Thoracic and supra-aortic trunk CTA with 3D volume rendering (**A**,**B**) and coronal reformatting (**C**) in a 41-year-old patient with TAK showing ectasias of the ascending and descending thoracic aorta (**A**), aneurysm of brachiocephalic artery (arrow in (**B**)) and stenoses of the common carotid arteries (arrows in (**C**)), as well as subclavian arteries.

In the acute stage of the disease, also called the “active stage,” CTA is often depicted by early changes in panarterial inflammation in the vessel wall and lumen, including circumferential vessel wall thickening (Figure 2), with subsequent stenosis and occlusion in almost all patients (Figure 3), as well as vessel ectasia and aneurysms (Figure 4) associated with thrombosis and ulcers in about a third of the patients [5,17,20,22]. The “string of pearls” sign can also be seen in some cases [17], with the frequent appearance of a “rat tail” revealing proximal dilation and distal tapering [13].

In cases of reduced inflammation, MRI and PET-CT can detect subtle changes, such as minimal inflammation of the arterial wall, that are not visible on CTA [5].

**Figure 5:**
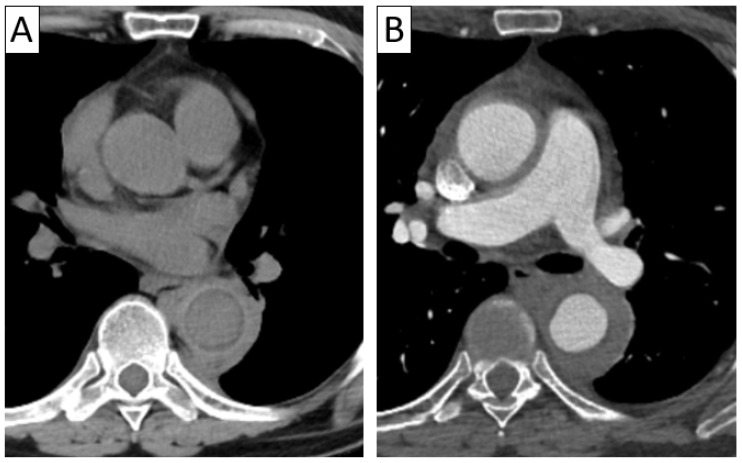
Contrast-enhanced axial thoracic CTA in a patient with TAK showing a highly attenuating thickened wall of the descending aorta on unenhanced scan (**A**) and the “double ring” sign on venous-phase imaging (**B**): a hypodense internal ring (intima) surrounded by an enhancing outer ring (media and adventitia).

Characteristically for early-stage TAK, on unenhanced CT, the thickened aortic wall is of high attenuation compared with the lumen, while on venous-phase images, a hypodense internal ring of swollen intima associated with an outer ring of enhancing media and adventitia gives the appearance of a “double ring” [5,20,21,23,24] (Figure 5).

In chronic stages of TAK, linear arterial wall calcification and collateral vessels can be seen indicative of longstanding “burned out aortitis” [5,20,21]. Characteristically, calcifications are usually transmural and not subintimal, as found in atherosclerosis [13].

Almost half of the patients diagnosed with TAK present with rapidly expanding aortic aneurysms, and up to a third of these aneurysms are complicated with ruptures and hemorrhages [20,24]. Minimally calcified aortas are more prone to aneurysmal dilatation [23].

Cardiac and coronary involvement has been described in both GCA and TAK with diffuse or focal coronary stenosis (Figure 3C) and aneurysms (“string of pearls” sign), valvular disease (aortic insufficiency, rarely mitral and tricuspid regurgitation), myocarditis, and pericarditis [17].

### 6.3. Pulmonary Involvement of LVV

Pulmonary vasculitides include disorders in which pathological changes in the vessel wall lead to the destruction of pulmonary blood vessels as a primary event or secondary to conditions such as connective tissue diseases, hypersensitivity disorders, infections, or malignancies [25,26].

Multidetector computed tomography (MDCT) angiography is the “work horse” in the radiologic assessment of pulmonary vasculitis. The key imaging feature of pulmonary LVV is the wall thickening of pulmonary arteries, which can progress to steno-occlusive disease with pulmonary oligemia and infarctions or it can lead to aneurysmal formation with a high risk of pulmonary hemorrhage. Secondary pulmonary hypertension is frequent in these cases and easily identifiable on MDCT [27].

#### 6.3.1. Behcet’s Disease

**Figure 6:**
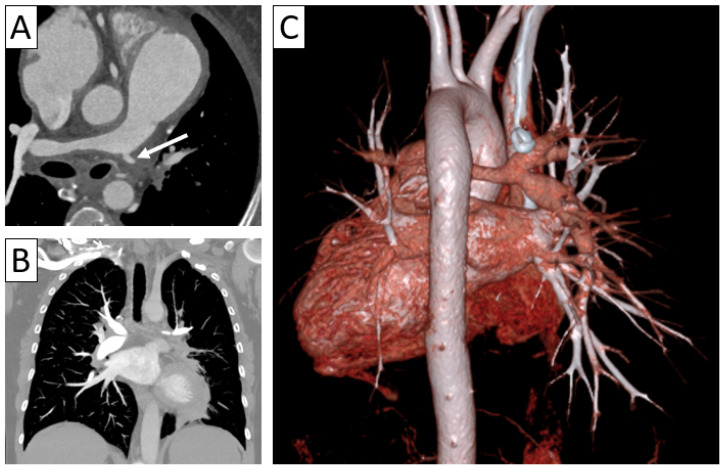
A 48-year-old female patient with Behcet’s disease presenting with oral aphthous ulcers and dyspnea. (**A**) Axial thoracic CTA showed diffuse thickening of the pulmonary trunk and arteries with left pulmonary artery stenosis (arrow) with extension to the lobar, segmental, and subsegmental left pulmonary arteries and subsequent vascular paucity and reduced volume of the left lung seen on coronal reformatting (**B**) and 3D volume rendering (**C**).

In BD, the association with large vessel lesions that occurs in 5–30% of cases [21] is referred to as vasculo-Behçet’s disease [25,28]. It classically presents as three entities: venous thrombosis (most frequent), arterial aneurysm, or occlusion (Figure 6) [21,28], as a result of inflammation of the vasa vasorum in the media and destruction of the elastic fibers of the vessel wall [25,28].

Aneurysms encountered in 65% of the patients with BD [28] can be located in the aorta, subclavian, or popliteal arteries. However, pulmonary artery aneurysms are commonly found, usually multiple and bilateral in the lower lobes or main pulmonary arteries, mostly saccular and fusiform types [21], with sizes of up to 7 cm [27]. BD is the most common cause of pulmonary artery aneurysms. Complication with hemoptysis is one of the leading causes of death: up to 30% in 2 years with a mean survival of 10 months from onset [25,28]. Unfortunately, the rapid evolvement of aneurysm size cannot be used to predict the risk of rupture [27]. CTA is the primary method of detecting aneurysms and for the characterization of related findings or therapy-induced changes [27]. Aneurysmal wall thickening usually denotes a subadventitial hematoma formation, while consolidation or ground-glass opacification of the perianeurysmal air space suggests impending rupture [27]. Partial or total in situ thrombosis of the aneurysm is frequently encountered [25]. It is worth mentioning that regression of aneurysms was preceded by thrombus formation, which also disappeared after immunosuppressant treatment [27].

Pulmonary arterial occlusion can be found in up to 35% of the patients with BD [28].

An important practical note is that angiography and venography should not be performed in patients with BD given the high risk of aneurysm development at the site of arterial puncture, as well as venous thrombosis after contrast material injection. Additionally, thrombosed aneurysms may not be visualized by angiography [28].

#### 6.3.2. Hughes–Stovin Syndrome

Hughes–Stovin syndrome (HSS) is a very rare disorder, an LVV characterized by multiple pulmonary and bronchial arterial aneurysms, as well as systemic venous thrombosis [5,29]. HSS, sometimes referred to as “incomplete Behçet’s disease,” presents many similarities with the manifestations of BD [5]: thrombosis of the vena cava, cerebral sinuses, or limb veins, as well as pulmonary arterial occlusions and aneurysms [27]. The absence of mucocutaneous changes can help differentiate the two entities [21].

**Figure 7:**
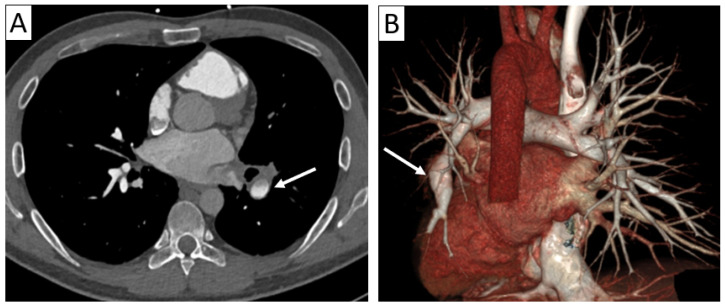
A 36-year-old male patient diagnosed with Hughes–Stovin syndrome presenting with hemoptysis and oro-genital aphthous ulcers with recurrent thrombotic events (inferior vena cava and renal veins). Axial thoracic CTA (**A**) with 3D-volume rendering (**B**) revealed a partially thrombosed left inferior lobar pulmonary artery aneurysm (arrows).

The main causes of vasculitic pulmonary aneurysms are BD and HSS (Figure 7).

Up to 50% of patients with HSS develop pulmonary arterial aneurysms and are at risk of hemorrhage [27]. In this sense, selective pulmonary angiography should be avoided as it carries the risk of aneurysmal rupture. The three main mechanisms of hemoptysis in HSS that can be objectified on CTA are rupture of a pulmonary artery aneurysm, active vasculitis with thrombosis, and bronchial artery hypertrophy secondary to ischemia of an occluded pulmonary artery [29].

#### 6.3.3. Takayasu Arteritis

Pulmonary arteritis with the development of aneurysm and stenoses can be found in 15% of patients with TAK [24].

Early stages of the disease present with vessel wall thickening and late enhancement as a primary event followed by luminal narrowing. Pulmonary artery involvement occurs in longstanding disease in about 50–80% of patients and usually manifests as stenosis or occlusion, with mural calcifications. In cases of pulmonary–systemic shunts or chronic pulmonary artery obstruction of unknown etiology, late-phase TAK should be considered as a differential diagnosis [25,26]. The “double ring” sign is not visible in the pulmonary arteries [27].

### 6.4. Differential Diagnosis and Mimickers

On imaging, LVV can mimic and even be mimicked by other entities. These burden the radiological diagnosis with multiple differential diagnostics and can even lead the interpretation and management of the case in a different and erroneous direction, sometimes posing life-threatening risks for the patient.

**Figure 8:**
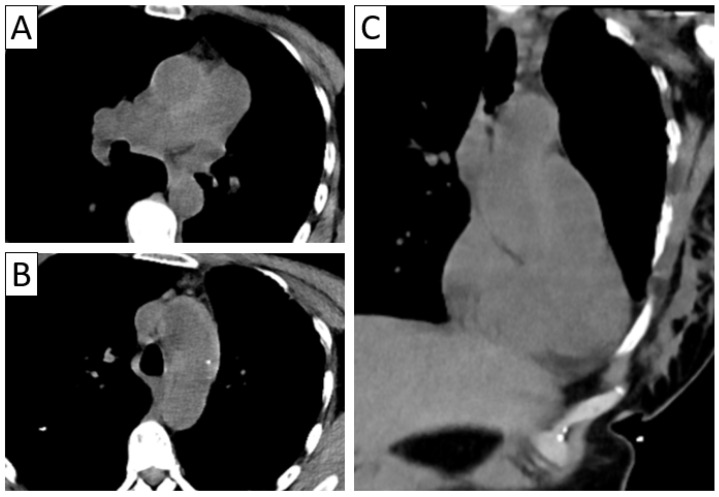
Differential diagnosis of vasculitis—intramural hematoma and pulmonary trunk sarcoma. Patient with sudden severe chest pain—unenhanced axial thoracic CT (**A**,**B**) with coronal reformatting (**C**) revealed arterial mural hyperdensities along the ascending aorta and pulmonary trunk suggestive of intramural hematoma; intraoperatively, suspicion of pulmonary trunk sarcoma was raised; histopathological result confirmed granulomatous necrotizing vasculitis.

Intramural hematoma. Unenhanced imaging, although sometimes overlooked, has the main benefit of differentiating the vessel wall thickening from intramural hematoma, an acute aortic syndrome [7]. On CTA, arterial wall inflammation has a low attenuation of under 40 HU on unenhanced images and late enhancement on post-contrast phases, whereas a higher attenuation of over 50 HU on both pre- and post-contrast imaging can be seen in cases of intramural hematoma [24]. Another useful characteristic is the extent of the changes along the circumference of the vessel: while vasculitis is circumferential, intramural hematoma classically presents as an eccentric semilunar hyperdensity, called the “high-attenuation crescent” sign [13,30] (Figure 8).

Surgical material. In some cases, the lack of experience in vascular and cardio-thoracic imaging, as well as the unavailability of previous surgical documentation [13], can lead to the misinterpretation of a vascular graft (prothesis of the ascending aorta or an aorto-iliac bypass, for example) that can present as a circumferential parietal higher attenuation on unenhanced scans [31]. Information about prior surgical interventions should always be available.

Atherosclerosis. Both LVV and atherosclerosis are characterized by inflammation of the arterial wall, to different degrees, with significant changes at the level of the intimal layer. Non-calcified atheromatous plaques are considered high-risk plaques and because they can sometimes appear as circumferential, differential diagnosis with focal vasculitic changes is important for the therapeutic approach. Patchy, multivascular involvement with irregular calcification can help aid the diagnosis [13,32].

Retroperitoneal fibrosis (chronic periaortitis or sclerosing retroperitoneal granuloma). Retroperitoneal fibrosis is a systemic disease in which there is retroperitoneal proliferation of fibrous tissue, which extends to the adjacent viscera (inferior vena cava and ureters). In over 70% of patients, it is idiopathic. However, in some cases, it can be related to inflammatory diseases, malignancies, or immunologic disorders [33]. Typically, it presents as a retroperitoneal paraspinal soft tissue mass that does not displace the great vessels [20].

**Figure 9:**
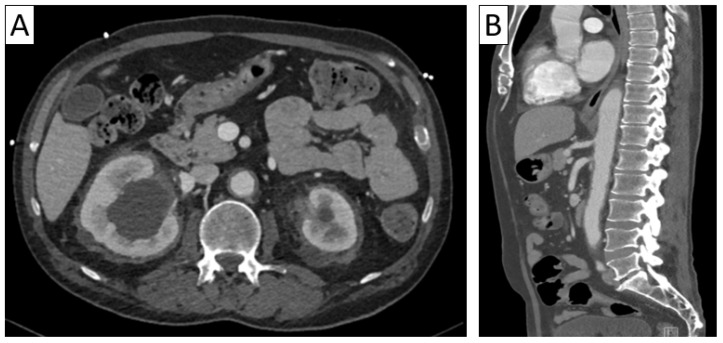
Differential diagnosis of vasculitis—Erdheim–Chester syndrome. Axial abdominal CT (**A**) with sagittal reformatting (**B**) showing diffuse retroperitoneal periaortic and perirenal tissue infiltration (“coated aorta” sign and “hairy kidneys”) in a patient with multiorgan affection (cerebral, osseous, renal, and pulmonary).

Erdheim–Chester syndrome. Erdheim–Chester syndrome, a non-Langerhans cell histiocytosis, is a rare systemic disease of unknown origin, characterized by tissue infiltration with foamy histiocytes (xanthogranulomatosis) [34], which presents with various manifestations: osteosclerosis of metaphysis and diaphysis the long tubular bones, cardiovascular and central nervous system involvement, and interstitial pulmonary diseases. Another frequent finding is retroperitoneal infiltration, which gives the characteristic signs of “hairy kidneys” and “coated aorta.” In these cases, CTA shows circumferential periaortic tissue infiltration that is perivascular (periadventitial) and not intramural [33,34] (Figure 9).

Acute pulmonary thromboembolism. Cases of BD with hemoptysis may be misdiagnosed as acute pulmonary thromboembolism given the frequent evidence of deep vein thrombosis [27]. However, deep vein thrombosis, although common in BD, rarely causes pulmonary embolism because the thrombi in the inflamed veins of the lower extremities are strongly adherent [28]. The presence of aneurysmal disease can aid the diagnosis [27].

Chronic thromboembolic pulmonary hypertension. TAK should be considered in cases of pulmonary arterial narrowing associated with constrictive wall thickening and should not be misinterpreted as chronic pulmonary thromboembolism [27], which is characterized by eccentric, sometimes calcified thrombi with complete or partial arterial obstruction, bands, webs, and poststenotic dilatation [35]. 

**Figure 10:**
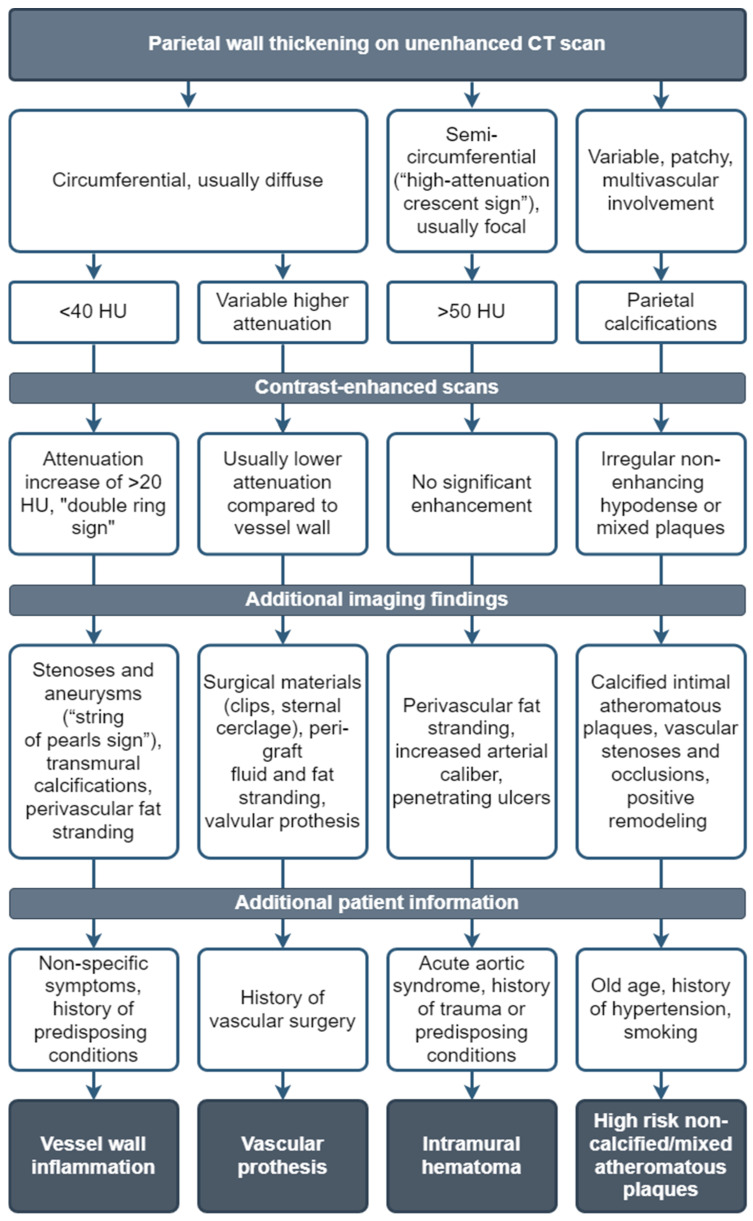
Suggested differential diagnosis workflow approach of the main mimickers of LVV.

Figure 10 presents the main mimickers of LVV, showcasing a suggested differential diagnosis workflow approach.

## 7. Multidisciplinary Approach to Large Vessel Vasculitis Management

A multidisciplinary approach is essential in the management of LVV, integrating the expertise of rheumatologists, radiologists, vascular surgeons, and other specialists, such as internal medicine doctors and pathologists, to ensure comprehensive care [36]. Rheumatologists typically lead the management of cases, overseeing the overall treatment strategy and coordinating with other specialists. Radiologists play a crucial role in the diagnosis, ongoing monitoring, and assessment of treatment response through different imaging techniques. Vascular surgeons may become involved when surgical interventions, such as angioplasty or stenting, are necessary due to complications like aneurysms or severe stenosis. This collaborative approach allows for timely and effective decision making, ensuring that each patient receives personalized and optimal care [2,36].

Imaging is crucial to the management of LVV, serving as a tool in both disease and treatment response monitoring. Regular imaging follow-ups enable clinicians to assess the effectiveness of treatment, guiding adjustments in therapy to prevent disease progression or relapse. Early detection of changes through imaging allows for prompt interventions.

Treatment adjustments and interventions are usually determined by clinical changes and imaging findings. Pharmacologically, corticosteroids are often the first line of treatment to rapidly control inflammation. Immunosuppressive agents are used to maintain remission and reduce the need for long-term corticosteroid use. Non-pharmacological strategies also play an important role; lifestyle modifications, including smoking cessation and blood pressure control, are essential in reducing cardiovascular risk factors. Vascular interventions, such as angioplasty or stenting, may be required in cases of severe vascular damage or complications [2,6]. The overall goal of the multidisciplinary team is to control the symptoms, prevent complications, and improve the quality of life for patients with LVV [2].

## 8. Future Directions

The topic of vasculitis has gained significant attention in recent years, yet the diagnosis and management of cases remain challenging. Through our work, we aim to ease the study of vasculitis and highlight key radiological findings, while sensitizing radiologists to the differential diagnoses of LVV.

One of the main limitations of our study is that, due to the complexity of vasculitides, we focused solely on LVV and included only CTA cases as illustrative examples. Similarly, while our work centers on the radiological aspects of LVV, the diagnosis may also require histopathological and genetic evaluations.

Currently, there is still room for innovation. Hybrid imaging techniques (PET) offer accuracy and clinical value and are expected to improve in the near future with important advancements, such as highly sensitive PET camera systems, PET/MRI, and dynamic (kinetic) imaging, providing a more comprehensive assessment of vascular inflammation, and more specific radiopharmaceuticals targeting immune cells and activated stromal cells [36].

Additionally, the application of artificial intelligence in image analysis could significantly improve the early detection of subtle vascular changes easily overlooked by radiologists. Recent accurate and robust models of artificial intelligence organ segmentation have been published, showing proficiency in anatomically delineating large vessels on CT scans on low-dose, non-contrast-enhanced CT images [36,37,38]. These technologies are expected to enhance the accuracy of disease monitoring and facilitate more precise assessments of treatment response.

The continued focus on personalized treatment strategies, guided by imaging and biomarker data, could potentially revolutionize patient care. By tailoring interventions to each patient’s specific needs, these approaches have the potential to significantly improve long-term outcomes and overall quality of life [36].

## 9. Conclusions

Advances in vascular imaging, including CTA, MRI, ultrasound, and PET-CT, have become essential in diagnosing and managing large vessel vasculitis. The diverse clinical manifestations and potential complications of vasculitis continue to pose significant challenges, and prompt identification of vasculitis is critical due to its association with high mortality risk. Imaging remains central to monitoring disease progression and treatment response, allowing for timely interventions. However, the risk of misdiagnosis owing to the potential of vasculitis to mimic other conditions underscores the need for careful interpretation and a multidisciplinary approach. Our work aims to highlight the importance of radiological expertise in improving the diagnosis and management of vasculitis, as well as enhance understanding and diagnostic accuracy among radiologists.

## Figures and Tables

**Table 1:** Vasculitis nomenclature adopted by the 2012 International Chapel Hill Consensus Conference on the Nomenclature of Vasculitides [4].

**Large vessel vasculitis**
Takayasu arteritis (TAK)
Giant cell arteritis (GCA)
**Medium vessel vasculitis**
Polyarteritis nodosa
Kawasaki disease
**Small vessel vasculitis**
Anti-neutrophil cytoplasmic antibody (ANCA)-associated vasculitis
Microscopic polyangiitis
Granulomatosis with polyangiitis (Wegener granulomatosis)
Eosinophilic granulomatosis with polyangiitis (Churg–Strauss syndrome)
Immune complex small vessel vasculitis
Anti-glomerular basement membrane (anti-GMB) disease
Cryoglobulinemic vasculitis
IgA vasculitis (Henoch–Schönlein purpura)
Hypocomplementemic urticarial vasculitis (anti-C1q vasculitis)
**Variable vessel vasculitis**
Behçet’s disease
Cogan syndrome
**Single-organ vasculitis**
Cutaneous leukocytoclastic angiitis
Cutaneous arteritis
Primary central nervous system vasculitis
Isolated aortitis
Others
**Vasculitis associated with systemic disease**
Lupus vasculitis
Rheumatoid vasculitis
Sarcoid vasculitis
Others
**Vasculitis associated with probable etiology**
Hepatitis C virus-associated cryoglobulinemic vasculitis
Hepatitis B virus-associated vasculitis
Syphilis-associated aortitis
Drug-associated immune complex vasculitis
Drug-associated ANCA–associated vasculitis
Cancer-associated vasculitis
Others

TAK—Takayasu arteritis, GCA—giant cell arteritis, ANCA—anti-neutrophil cytoplasmic antibody; GMB—glomerular basement membrane.

**Table 2:** Main advantages and disadvantages of different imaging modalities in the diagnosis of LVV. Adapted after Schäfer et al. [11] and Dejaco et al. [12].

Imaging Modality	Advantages	Disadvantages
**US**	Easily accessible Comfortable for patients Brief acquisition time of around 15 min Suitable for fast-track clinics More cost-effective Very high resolution (up to 0.1 mm in superficial anatomical structures) Strong evidence	Assessment of the thoracic and abdominal aorta is limited Overview of involved vessels is limited
**CTA**	Comprehensive overview of the aorta and its branches Different contrast phases for assessment Clear delineation of atherosclerosis Relatively quick acquisition time	Irradiation of about 17 mSv Contrast agent usage limited by reduced kidney function
**MRA**	Identifies characteristic arterial abnormalities Excellent overview of affected arteries Simultaneous assessment of cranial and extracranial arteries Better visualization of the aorta compared to US No irradiation No iodinated contrast agents	Lower sensitivity compared to CT and US for detecting calcifications More costly than US Claustrophobia Contraindications (cardiac pacemakers or other implanted metal devices) Long acquisition time Limited expertise
**PET/CT**	Good overview of affected arteries Ability to detect differential diagnoses of LVV like infections or malignancies Potentially more sensitive than MRI in detecting disease activity Strong evidence level in LVV	Irradiation of about 25 mSv Costly procedure Not possible in elevated glucose levels Sensitivity significantly decreases after more than 3 days Atherosclerosis, especially in femoral arteries, may be mistaken for LVV

US—ultrasound, CTA—computed tomography angiography, CT—computed tomography, MRA—magnetic resonance angiography, MRI—magnetic resonance imaging, PET/CT—positron emission tomography-computed tomography, LVV—large vessel vasculitis, mSv—millisievert.

**Table 3:** Imaging findings in primary LVV. Adapted after Aghayev et al. [7].

Primary Vasculitis	GCA	TAK
**US**	“Halo sign” (circumferential hypoechoic rim around the vessel lumen on transversal view)—temporal artery and extremity vessels Positive “compression sign” (no collapse of the inflamed vessel which is still visible after compression)	“Halo sign”—cervical and extremity vessels
**CTA**	Circumferential parietal thickening Vessel wall enhancement	Circumferential parietal thickening Vessel wall enhancement Luminal stenosis or narrowing
**MRA**	Circumferential parietal thickening Vessel wall enhancement Wall enhancement of superficial cranial arteries (on high-resolution MRI)	Circumferential parietal thickening Vessel wall enhancement Luminal stenosis or narrowing
**PET/CT**	Segmental FDG uptake of vessels equal to or more than the liver FDG uptake of shoulder/hip joints or synovium (if associated with Polymyalgia rheumatica)	Segmental FDG uptake of vessels equal to or more than liver

GCA—giant cell arteritis, TAK—Takayasu arteritis, US—ultrasound, CTA—computed tomography angiography, MRA—magnetic Resonance Angiography, MRI—magnetic resonance imaging, PET/CT—positron emission tomography-computed tomography, FDG—fludeoxyglucose-18.
